# The effect of image-guided radiation therapy on the margin between the clinical target volume and planning target volume in lung cancer

**DOI:** 10.1002/jmrs.42

**Published:** 2014-02-26

**Authors:** Jun Liang, Minghui Li, Tao Zhang, Wei Han, Dongfu Chen, Zhouguang Hui, Jima Lv, Zhong Zhang, Yin Zhang, Liansheng Zhang, Rong Zheng, Jianrong Dai, Luhua Wang

**Affiliations:** 1 Department of Radiotherapy, Cancer Hospital, Chinese Academy of Medical SciencesBeijing, China

**Keywords:** Image-guided radiotherapy, lung neoplasm, set up error, target volume

## Abstract

**Introduction**This study aimed to evaluate the effect of image-guided radiation therapy (IGRT) on the margin between the clinical target volume (CTV) and planning target volume (PTV) in lung cancer.

**Methods**The CTV and PTV margin were determined in three dimensions by four radiation oncologists using a standard method in 10 lung cancer patients, and compared to consensus values. Transfer error was measured using a rigid phantom containing gold markers. Systematic error (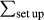) and random error (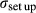) set up errors were calculated in three dimensions from pre-treatment and post-treatment cone beam CT scans. Finally, the margin between the CTV and PTV was corrected for set up error and calculated.

**Results**The margins between the CTV and PTV with IGRT (and without IGRT) were 0.88 cm (0.96 cm), 0.99 cm (1.08 cm) and 1.28 cm (1.82 cm) in the anterior and posterior (AP), left and right (LR) and superior and inferior (SI) directions, respectively. Images from two other patients verified the validity of the corrected margin. The target delineation errors of the radiation oncologists are considered to be the largest compared with the set up errors. The application of IGRT reduced the set up errors and the margins between CTV and PTV.

**Conclusions**The delineation errors of radiation oncologists are the most important factor to consider for the margin between CTV and PTV for lung cancer. IGRT can reduce the margins by reducing the set up errors, especially in the SI direction. Further research is required to assess whether the reduction in the margin is solely based on set up errors.

## Introduction

Radiotherapy (RT) is one of the most effective modalities for the treatment of cancer; however, it can take weeks or even months and requires several steps from the decision to treat with RT to completing treatment. Potential errors can occur at each step. During the target definition step, sources of uncertainties include: target motion, patient set up errors, organ (tumour) motion and delineation of the target volume(s).^1^

According to the nomenclature of the International Commission on Radiation Units and Measurements Report 50, macroscopic disease, which is apparent on clinical examination or imaging, is defined by the gross tumour volume (GTV). The clinical target volume (CTV) incorporates this volume and adds the volumes in which the disease is expected to be microscopically present based upon the natural history of the disease and relapse patterns. The planning target volume (PTV) is an enlargement of the CTV, which takes into account the internal motion of the target volume and set up variations.^2^

Imaging techniques have been introduced not only at this stage but also at the treatment stage, and can control patient set up errors and (or) organ motion. RT treatment delivery using these techniques is collectively called image-guided radiation therapy (IGRT). IGRT provides an essential tool to investigate, quantify and in turn correct for geometrical uncertainties.^3^

The three main reasons for local failure after RT in lung cancer are: (1) geographical misses due to the inadequacy of imaging tools for staging and RT planning, (2) geographical misses due to respiratory tumour motion during RT delivery and (3) inadequate RT doses due to the potential for significant toxicity.^4^ IGRT enables more accurate tumour targeting, thereby reducing side effects and (or) providing the opportunity to escalate the tumour dose. Cone beam computed tomography (CBCT) is the major type of IGRT used for the treatment of lung tumours, as it provides excellent visualisation of the target with volumetric imaging and relatively high-resolution soft-tissue information. Studies have revealed that CBCT-based IGRT significantly reduces the set up margin in lung cancer. Yeung et al. found that CBCT-based IGRT reduced the set up margin by up to ∼1.5 cm, compared with no image guidance or image guidance using bony anatomy as a surrogate for the target(s) in lung cancer.^5^ Ottosson et al. reported that the margin can be reduced by half when CBCT is used to correct set up errors in lung cancer.^6^

The definition of set up errors is errors that relate to the treatment position of the patient and are fixed for each treatment course. Reduction in set up errors by IGRT may reduce the margin between the CTV and PTV. However, there are many factors influencing the margin between the CTV and PTV, so is it reasonable to reduce this margin based only on set up errors? The aim of this study was to evaluate the effect of IGRT on the margin between the CTV and PTV in lung cancer. In this study, target volume delineation, target motion from respiration, phantom transfer errors and set up errors are considered as influencing factors. Organ position and shape at time of localisation, which comprise geometric imaging errors, are not considered.

## Methods

This study protocol was approved with the approval number 13-075/751 by the institutional ethical review committee and permission to conduct the study was granted by the institutional review board. The study has been registered according to the Good Clinical Practice of the Cancer Institute and Hospital, Chinese Academy of Medical Sciences.

The margins between the CTV and PTV in lung cancer were calculated following the guidelines in Geometric Uncertainties in RT.^7^ The factors influencing the margin include: (1) uncertainties of target-volume delineation; (2) physiological changes, such as target motion from respiration; (3) phantom transfer errors, which accumulate during transfer of the imaging data from the initial localisation images through the treatment planning system to the linear accelerator and can be measured with a phantom containing gold markers; and (4) set up errors and other factors.^8^

### Inter-observer uncertainties in delineation of the target volume

#### Simulation

Ten patients were positioned supine on the couch of a CT simulator (Philips Inc., Cleveland, OH) and immobilised with thermoplastic masks. For each patient, the localisation centre was marked on the mask. Transverse images were scanned for the body/chest from the mandible to the second lumbar vertebra at a 3-mm slice thickness. Positron emission tomography (PET) is important for accurate target delineation in lung cancer.^9^,^10^ Therefore, the patients also received a PET-CT scan in the same position (GE, Discovery ST16; GE Inc., Waukesha, WI). Both the simulation CT images and PET-CT images were transferred to the treatment planning system through the network (Philips Pinnacle 3).

#### Delineation of GTV and CTV

Four radiation oncologists that have been trained for target volume delineation and have worked for more than 15 years independently defined the GTV and CTV for the 10 patients on a treatment planning system (Philips Pinnacle 3 version 6.3). Many studies show that radiation oncologists choose a value or threshold value based on the PET standard uptake value (SUV).^11^ Some authors have suggested the use of percentage values for the maximum SUV of the primary tumour, while others have suggested a SUV of 2.5,^12^ which was used in this study.

The GTV of non-small cell lung cancer (NSCLC) included the primary tumour and lymph nodes with metastases which showed on the images (reference CT, PET, fibreoptic broncoscopy [FOB]). The GTV was delineated on the CT images using the PET scan as a reference. CTV was defined as the GTV with a 6-mm margin for squamous cell carcinoma, the GTV with an 8-mm margin for adenocarcinoma, or a 1.5-cm margin of the main trachea in central lung cancer.^13^,^14^ The CTV was not to exceed the delineated anatomical margin, except for invasion. For prophylactic regional lymph node irradiation, the CTV included the ipsilateral pulmonary hilar region if mediastinal lymph node metastases were present. Additionally, the CTV included the subcarinal region if mediastinal lymph node metastases were detected in the right middle or lower lobe, left ligule or lower lobe. Otherwise, the CTV included the subaortic region if left upper mediastinal lymph node metastases were present in left upper lobe cancer. In small cell lung cancer (SCLC), the GTV was defined as the primary tumour after chemotherapy, and the CTV was defined as the GTV with an 8-mm margin and the regional lymph nodes metastases detected before chemotherapy.

#### Data analysis

**Figure 1:**
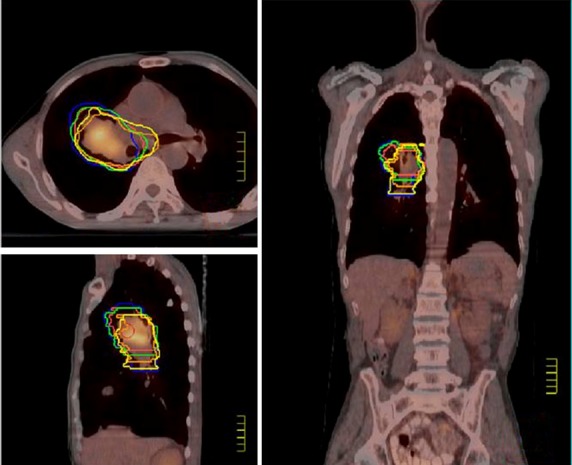
The yellow line represents the clinical target volume (CTV) CTV approved by consensus.

The CTV values determined by each observer were compared to the CTV values approved by consensus in three dimensions (Fig. 1). The inter-observer CTV delineation errors (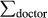) were calculated using the equation:


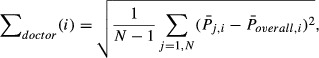
(1)

where *i* is the direction index, *j* is the patient index, *N* is the total number of patients, 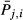 is the average unilateral delineation difference of patient *j* in direction *i*, and 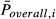 is the average unilateral delineation difference of all patients in direction *i*.

### Target motion from respiration

Three methods are used to deal with target motion due to respiration at the Cancer Institute and Hospital, Chinese Academy of Medical Sciences: observation of the primary tumour motion using a conventional simulator, a four-dimensional CT simulator or a PET-CT simulator. Target motion can be explicitly determined using a conventional simulator or a four-dimensional CT simulator; therefore, the margin formula includes a component to represent the contribution of target motion. PET images are acquired over minutes or even tens of minutes and target motion is reflected as an enlarged target in the images; therefore, the contribution of target motion is not included in the margin formula. In this study, all 10 patients received PET-CT simulation; therefore, target motion was not included in the formula to calculate the margin.

### Phantom transfer error

RT includes three basic steps: simulation, planning and delivery; with different equipment required for each step (simulator for simulation, planning system for planning and treatment machine for delivery). Each machine has its own coordinate system, which should be identical. However, minor differences exist between the simulator and treatment machine due to limitations in the accuracy of manufacturing. Such differences result in the transfer error, which can be measured using a rigid phantom.

### Set up errors

Slight differences occurred in the positioning and immobilisation of patients for each treatment fraction and between treatments. These errors are set up errors which relate to the treatment position and fixation. The 10 patients studied received pre-treatment and post-treatment cone beam CT scans on the Elekta Synergy linear accelerator, with 72 and 66 image series acquired and analysed respectively. The characteristics of the 10 patients are shown in Table 6. The systematic set up error (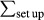) in three dimensions was calculated as:


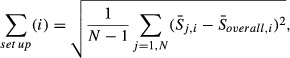
(2)

where 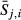 is the average set up error for patient j in direction *i*, and 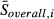 is the overall mean population set up error for all patients. The random set up error (*σ*_set up_) in three dimensions was calculated using the equation:


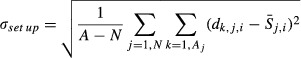
(3)

where *k* is the set up index, *A* is the set up error summation for all patients, d*k*,*j*,*i* is the *k* set up error for patient *j* in direction *i* and 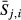 is the average set up error for patient *j* in *i* direction.

### Calculation of the CTV and PTV margin corrected for set up errors

The margin between the CTV and PTV corrected for set up errors was calculated using the following:


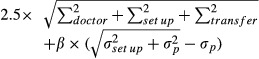
(4)

where *β* is a parameter related to the beam configuration, and *σ*_*p*_ is the photon penumbra width. The values for *β* and *σ*_*p*_ were adopted from Holmberg et al.^7^

## Results

The differences between the delineations of the CTV of individual radiation oncologists are shown in Figure 1 and Table 1. The differences in the delineation error of individual doctors (∑ _*doctor*_) was 0.32 cm in the anterior and posterior (AP, Z) direction, 0.37 cm in the left and right (LR, X) direction and 0.48 cm in the superior and inferior (SI, Y) direction. Table 2, 3 show the pre-treatment and post-treatment set up errors during CBCT in three dimensions. Table 4 shows the random error (*σ*_*set up*_) and system error (∑ _*doctor*_) set up errors in three dimensions.

**Table 1:** Inter-observer differences in clinical target volume (CTV) CTV.

	Patient number					
	1		2		3	
	Anterior (A)-posterior (P) direction (cm)					
	A	P	A	P	A	P
Doctor 1	−0.293	0.195	−0.098	0.195	0.274	0.293
Doctor 2	−0.195	−0.391	−0.488	0.683	0.137	−0.39
Doctor 3	−0.293	0.391	0.000	−0.098	0.547	0.586
Doctor 4	−0.39	0.098	−0.488	0.879	0.274	−0.292
Average	−0.292	0.073	−0.268	0.414	0.308	0.078
Unilateral average	−0.109		0.073		0.193	
	Left (L)-right (R) direction (cm)					
	L	R	L	R	L	R
Doctor 1	1.172	0.097	−0.097	−0.976	0.293	−0.195
Doctor 2	0	0.488	0.098	−0.976	0.196	0.391
Doctor 3	0.586	−0.489	−0.097	−0.586	0.098	0.293
Doctor 4	0.098	1.757	0	−0.097	0.684	0.391
Average	0.464	0.463	−0.024	−0.658	0.317	0.22
Unilateral average	0.463		0.317		0.268	
	Superior (S)-inferior (I) direction (cm)					
	S	I	S	I	S	I
Doctor 1	0.000	2.289	−0.327	0.981	−0.654	−0.654
Doctor 2	0.327	0.327	0.654	0.654	0.327	0
Doctor 3	0.327	0.327	0.346	0.327	0.654	−1.327
Doctor 4	0.327	0	0.327	0.654	0	0
Average	0.245	0.735	0.134	0.588	0.082	−0.495
Unilateral average	0.49		0.361		−0.206	

**Table 2:** Pre-treatment set up errors during cone beam computed tomography (CBCT).

		Number of patients					
pre-treatment	(cm)	1	2	3	4	5	6
Anterior–posterior	Average	0.12	0.04	−0.33	−0.16	−0.17	−0.16
	SD	0.28	0.49	0.25	0.18	0.24	0.60
Left–right	Average	−0.65	−0.27	0.64	0.10	−0.25	−0.06
	SD	0.24	0.26	0.48	0.46	0.28	0.25
Superior–inferior	Average	1.25	0.36	0.06	0.37	0.71	−0.15
	SD	0.53	0.62	0.49	0.39	0.42	0.58

**Table 3:** Post-treatment set up errors during cone beam computed tomoraphy (CBCT).

		No. patients					
pre-treatment	(cm)	1	2	3	4	5	6
Anterior–posterior	Average	−0.02	−0.04	−0.19	0.07	−0.01	−0.25
	SD	0.14	0.13	0.14	0.08	0.11	0.04
Left–right	Average	−0.05	0.03	−0.17	0.07	0.01	0.14
	SD	0.12	0.12	0.19	0.10	0.13	0.08
Superior–inferior	Average	−0.05	−0.01	−0.01	0.06	0.09	−0.07
	SD	0.22	0.12	0.19	0.18	0.18	0.13

**Table 4:** Post-treatment and pre-treatment random errors (σ) and system errors (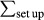) in three dimensional directions.

	Post-treatment (pre-treatment) error		
	AP (Z)	LR (X)	SI (Y)
∑_*set up*_	0.10 (0.14) cm	0.11 (0.16) cm	0.09 (0.33) cm
*σ*_*set up*_	0.12 (0.29) cm	0.13 (0.31) cm	0.17 (0.51) cm

AP, anterior–posterior; LR, left–right; SI, superior–inferior.

The systematic error (∑ _*transfer*_) of the reference frame was 0.10 cm in the AP (Z) direction, 0.07 cm in the LR (X) direction and 0.08 cm in the SI (Y) direction.

Table 5 lists the values for the corrected CTV and PTV margins, which were calculated using formula 4. The margins with and without set up correction were 0.88 cm and 0.92 cm in the AP direction, 0.99 cm and 0.99 cm in the LR direction and 1.28 cm and 1.43 cm in the SI direction, respectively.

**Table 5:** Values used for calculation of the post-treatment and pre-treatment CTV and PTV margins.

	Post-treatment (pre-treatment) values		
Directions	AP (Z)	LR (X)	SI (Y)
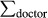	0.32 scm	0.37 cm	0.48 cm
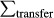	0.10 cm	0.07 cm	0.08 cm
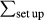	0.10 (0.14) cm	0.11 (0.16) cm	0.09 (0.33) cm
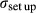	0.12 (0.29) cm	0.13 (0.31) cm	0.17 (0.51) cm
*σ*_*p*_	0.5 cm	0.5 cm	0.5 cm
*β*	0.67	0.67	1.64
Margin	0.88 (0.96) cm	0.99 (1.08) cm	1.28 (1.82) cm

CTV, clinical target volume; PTV, planning target volume; AP, anterior–posterior; LR, left–right; SI, superior–inferior.

### Verification of the validity of the corrected CTV and PTV margin

**Figure 2:**
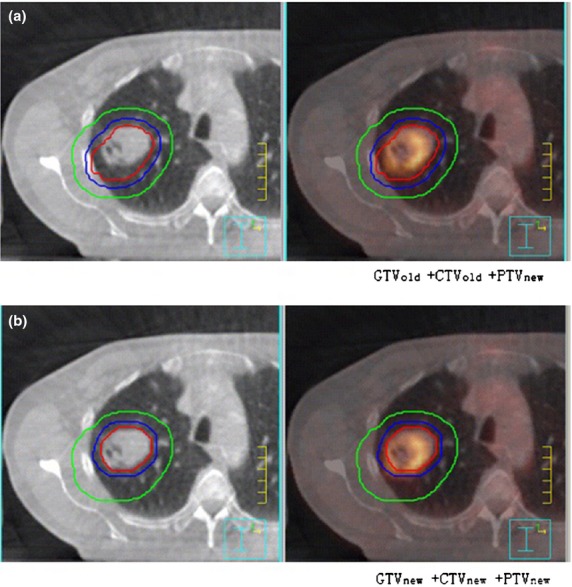
Verification of the margin between the CTV and PTV corrected for set up errors in (A) GTVold + CTVold +PTVnew and (B) GTVnew + CTVnew + PTVnew. The red line indicates the GTV, the blue line indicates the CTV and the green line indicates the PTV. CTV, clinical target volume; PTV, planning target volume; GTVold, old gross tumour volume; CTVold, old clinical target volume; PTVnew, new planning target volume; GTVnew, new gross tumour volume; CTVnew, new clinical target volume; GTV, gross tumour volume.

Two patients who received PET-CT localisation and IGRT were selected for further analysis. The margin values from CTVold to PTVnew were set according to Table 5. The post-treatment CBCT images from the first 3 weeks were retrieved and transferred to the planning system. The CBCT images and CT images were registered, then CTVnew was delineated on the CBCT images to observe the relationship between CTVnew and PTVnew. Figure 2 illustrates that all of the CTVnew was included in the PTVnew.

## Discussion

It can be difficult to decide the margin between the CTV and PTV in lung cancer, as there are many influencing factors and complicated formulae. From Table 5, the target volume delineation errors of radiation oncologists were the largest at 0.32 cm in AP, 0.37 cm in LR and 0.48 cm in SI. The set up errors with IGRT were 0.1 cm in AP, 0.11 cm in LR, and 0.09 cm in SI, which were approximately a third of the delineation errors. Therefore, it is likely that the most important factor for the margin between CTV and PTV is the target volume delineation errors of doctors, whereas the impact of set up errors is small. Even though all of our radiation oncologists have been trained, use the same delineation protocol and have more than 15 years of experience, delineation errors still existed.

Herk et al. advised that systematic errors introduced by target volume delineation, organ motion, and set up errors have a similar magnitude and should be reduced by clear delineation protocols, multimodality imaging, correct CT scan procedures and electronic portal imaging with decision rules.^15^ Technological advances in treatment planning algorithms, radiotherapy delivery, motion management and patient set up have considerably improved the precision of RT; however, as precision improves, errors in target delineation take on greater levels of importance. The impact of target volume delineation error may be a significant source of error in addition to other classic sources, such as the set up, inter-fractional or intra-fractional errors in the IGRT era. The target volume delineation variation depends on the target, the surrounding tissue, the imaging modality, the observers and the delineation protocol. Steenbakkers et al. demonstrated that delineation accuracy can be improved with the use of a standardised delineation protocol in addition to PET information. The reduction in interpretation differences among radiation oncologists by using PET/CT caused the largest improvement in delineation accuracy. However, even with the proposed improvements, observer variation in delineation was still a major source of geometric error in external beam RT.^16^ This finding was also demonstrated by this study.

The application of IGRT in this study reduced the set up errors from 0.14 cm to 0.1 cm in AP, 0.16 cm to 0.11 cm in LR and from 0.33 cm to 0.09 cm in SI. These results contributed to the margin reduction which fell from 0.96 cm to 0.88 cm in AP, 1.08 cm to 0.99 cm in LR and from 1.82 cm to 1.28 cm in SI. The margin values were largest in the SI direction, which may be related to differences in the delineation by individual radiation oncologists and the spacing of the CT image slices. The LR differences were related to regional lymph node involvement. The application of IGRT reduced the margin between the CTV and PTV in all three directions, especially SI, which decreased by 0.54 cm. These results show that IGRT could reduce the margin between the CTV and PTV of lung cancer, but the largest effect was in the SI direction. The GTV of all 10 patients was less than 90 cm^3^, except for one patient (141 cm^3^). Most patients received IGRT at the first three irradiations, and subsequent set up errors were corrected; therefore, the reported set up errors are smaller than the other errors.

Herk et al. recommended the formula (5) to calculate the margin from CTV to PTV.^17^


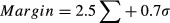


This margin formula takes into account the errors; in this formula, ∑ is total standard deviation (SD) of systematic error and *σ* is total SD of random error. However, this formula was not created for individual cancers. The target delineation error provided in their study was from prostate cancer, and was smaller than that of this study. They show that the set up error was the major factor, and systematic and random errors mostly resulted from set up errors. They proposed that this formula should be considered as a lower limit for the administration of safe RT. Even if the values are close to our results, the definitions would be different between cancers. This might explain why the results we obtained for lung cancers were different to those of their study.

## Conclusions

The target volume delineation errors of radiation oncologists are the most important factor for determining the margin between the CTV and PTV for lung cancer. IGRT can reduce the margin by reducing the set up errors, especially in the SI direction. However, further research is required to confirm that the reduction in the margin is solely based on set up errors.

## Conflict of Interest

None declared.

## APPENDIX

**A1:** Characteristics of the 10 lung cancer patients

Patient no.	Diagnosis	CTV (including GTV and mediastinal lymph node regions)	GTV(cm^3^)	Total dose/dose per fraction/number of fractions	No. of CBCT before regulation	No. of CBCT after irradiation
1	Right upper lobe adenocarcinoma post operation and chemotherapy right pulmonary hilar metastasis lymph nodes	10R	9.64	60 Gy/3 Gy/20f	11	10
2	Right upper lobe squamous carcinoma	GTV, 2R, 4R, 7, 10R	20.55	66 Gy/2 Gy/33f	7	7
3	Right upper lobe squamous carcinoma superior vena cava syndrome	GTV, 2R, 4, 5, 10R	62.45	66 Gy/2 Gy/33f	9	9
4	Right lower lobe NSCLC	GTV, 7, 10R	81.08	60 Gy/2 Gy/30f	7	7
5	Right upper lobe cancer	GTV	21.97	60 Gy/6 Gy/10f	10	9
6	Left upper lobe squamous carcinoma	GTV, 4L, 5, 6, 7, 10L	140.76	58 Gy/2 Gy/29f	4	4
7	Right lobe SCLC post operation and chemotherapy metastasis mediastinal lymph nodes	4R, 7	5.28	60 Gy/2 Gy/30f	9	9
8	Left lower lobe squamous carcinoma post chemotherapy	GTV, 2, 4, 7, 10L	44.23	60 Gy/2 Gy/30f	4	4
9	Left upper lobe SCLC post chemotherapy lung metastasis	GTV, 6, 5, 10L	88.58	37.5 Gy/2.5 Gy/15f	5	4
10	Right lower lobe squamous carcinoma	GTV, 7, 10R	81.15	60 Gy/2 Gy/30f	7	7

CTV, clinical target volume; GTV, gross tumour volume; NSCLC, non-small cell lung carcinoma; SCLC, small cell lung carcinoma; R, right; L, left; Gy, Gray; f, fractions; CBCT, cone beam computed tomography.
